# Antimicrobial Consumption among 66 Acute Care Hospitals in Catalonia: Impact of the COVID-19 Pandemic

**DOI:** 10.3390/antibiotics10080943

**Published:** 2021-08-04

**Authors:** Santiago Grau, Sergi Hernández, Daniel Echeverría-Esnal, Alexander Almendral, Ricard Ferrer, Enric Limón, Juan Pablo Horcajada, on behalf of the Catalan Infection Control Antimicrobial Stewardship Program (VINCat-PROA)

**Affiliations:** 1 Department of Pharmacy, Hospital del Mar, Passeig Maritim 25–29, 08003 Barcelona, Spain; dechevarria@psmar.cat; 2 Infectious Pathology and Antimicrobials Research Group (IPAR), Institut Hospital del Mar d’Investigacions Mèdiques (IMIM), Dr. Aiguader 88, 08003 Barcelona, Spain; jhorcajada@psmar.cat; 3 Department of Pharmacology, Universitat Autònoma de Barcelona, 08193 Barcelona, Spain; 4 VINCat Program Surveillance of Healthcare Related Infections in Catalonia, 08907 Barcelona, Spain; shernandezbaeza@iconcologia.net (S.H.); alexanderalmendral@iconcologia.net (A.A.); elimon@iconcologia.net (E.L.); 5 Department of Intensive Care Medicine, Hospital Universitario Vall D’Hebron, Passeig de la Vall D’Hebron 119, 08035 Barcelona, Spain; r.ferrer@vhebron.net; 6 Infectious Diseases Department, Hospital del Mar, Passeig Maritim 25–29, 08003 Barcelona, Spain; 7 Department of Medicine, CEXS-Universitat Pompeu Fabra, 08003 Barcelona, Spain

**Keywords:** antimicrobial consumption, COVID-19, defined daily doses, antibiotics, antifungals

## Abstract

Background: Antimicrobials have been widely used during the COVID-19 pandemic. This study aimed to analyze the impact of the COVID-19 pandemic on the antimicrobial consumption of 66 hospitals in Catalonia. Methods: Adult antibacterial and antimycotic consumption was calculated as defined daily doses (DDD)/100 bed-days and DDD/100 discharges. Firstly, overall and ICU consumption in 2019 and 2020 were compared. Secondly, observed ICU 2020 consumptions were compared with non-COVID-19 2020 estimated consumptions (based on the trend from 2008–2019). Results: Overall, antibacterial consumption increased by 2.31% and 4.15% DDD/100 bed-days and DDD/100 discharges, respectively. Azithromycin (105.4% and 109.08% DDD/100 bed-days and DDD/100 discharges, respectively) and ceftriaxone (25.72% and 27.97% DDD/100 bed-days and DDD/100 discharges, respectively) mainly accounted for this finding. Likewise, antifungal consumption increased by 10.25% DDD/100 bed-days and 12.22% DDD/100 discharges, mainly due to echinocandins or amphotericin B. ICU antibacterial and antimycotic consumption decreased by 1.28% and 4.35% DDD/100 bed-days, respectively. On the contrary, antibacterial and antifungal use, expressed in DDD/100 discharges, increased by 23.42% and 19.58%. Azithromycin (275.09%), ceftriaxone (55.11%), cefepime (106.35%), vancomycin (29.81%), linezolid (31.28%), amphotericin B (87.98%), and voriconazole (96.17%) use changed the most. Observed consumption of amphotericin B, azithromycin, caspofungin, ceftriaxone, vancomycin, and voriconazole were higher than estimated values. Conclusions: The consumption indicators for most antimicrobials deviated from the expected trend pattern. A worrisome increase in antibacterial and antifungal consumption was observed in ICUs in Catalonia.

## 1. Introduction

Severe acute respiratory syndrome coronavirus 2 (SARS-CoV-2) causes the coronavirus-disease 19 (COVID-19) [1]. This virus has caused the worst pandemic in the last century, posing a global threat [1]. At the health level, this pandemic has placed an unsustainable pressure on hospital systems, and especially on intensive care units (ICUs) [2,3]. The worldwide impact of the COVID-19 pandemic has been devastating, with more than 177,866,160 cases and 3,857,974 deaths, as of 24 June 2021 [4].

COVID-19 is characterized by a first phase with typical viral symptoms, followed by an inflammatory phase [5]. While 80% of patients present a mild clinical course, 5–14.2% require ICU admission and 6.1–12.2% require mechanical ventilation [6,7].

Bacterial and fungal co-infections and secondary infections are common in viral infections and are associated with increased morbidity and mortality [8]. In hospitalized patients infected with influenza, 23% present a bacterial co-infection, mainly due to *Staphylococcus aureus* or *Streptococcus pneumoniae* [9]. In other viruses, this rate is 40% [10]. Based on these data, at the beginning of the COVID-19 pandemic, antibiotics were widely used. However, subsequent studies demonstrated that the bacterial infection rate was lower than expected, with an incidence of 5.9% in hospital wards and 8.1% in ICUs [2], most of them due to *S. aureus* and *Haemophilus influenzae* [8]. Despite this information, the prevalence of antibiotic prescriptions was 74.6–85.2% [8,11]. Since then, international guidelines have accordingly adapted their recommendations, reserving the use of antibiotics for critically ill patients, or when a high clinical suspicion of bacterial infection exists [12].

This overuse of antibiotics may worsen the other pandemic of our century—that of global bacterial resistance [2,8]. In this setting, antimicrobial stewardship teams are indispensable to optimize antibiotic prescription and decrease the spread of multidrug-resistant organisms [2,11]. A knowledge of local consumption patterns is essential to identify opportunities and implement antibiotic policies that optimize their prescription. In the case of COVID-19, this information is crucial to learn from possible errors and avoid antibiotic misuse, both in further waves and other viral pandemics, which seem inevitable.

Antimicrobial consumption during the COVID-19 pandemic has previously been described [3,8,13,14,15]. However, most of these studies have been of single centers, with small sample sizes, and have not included the consumption indicator defined daily doses (DDD)/100 discharges or have not focused on the consumption in the ICU or the consumption of antifungals.

Catalonia, a Spanish region of 7.5 million inhabitants, developed the Catalan Infection Control Program (VINCat) 14 years ago. This program is a healthcare-associated infection surveillance program, which includes several objectives such as the knowledge of the incidence of nosocomial infections, catheter-related infections, and surgical wound infections [16]. One of the goals of this program is to control the use of antimicrobials in hospitals in this region through the extension of the implantation of antimicrobial stewardship programs (VINCat-PROA) in these centers, to later be extended to all areas of antimicrobial use. For that purpose, antimicrobial consumption is monitored every year.

The objective of this study was to analyze the impact of the COVID-19 pandemic in the antimicrobial consumption of hospitals included in the VINCat-PROA program, with emphasis on the ICU.

## 2. Results

### 2.1. Evolution of COVID-19 Pandemic in Catalonia during 2020

The first COVID-19 case in Catalonia was diagnosed on 25 February 2020. Since then, a total of 356,724 positive cases were diagnosed in 2020, with a mean effective reproduction number of 0.8 and a mean 14 day incidence of 1006.79 cases per 100,000 inhabitants [17,18]. A total of 8723 deaths were notified, with 29,773 hospitalizations and 2426 ICU admissions [18].

### 2.2. Evolution of Overall and ICU Bed-Days and Discharges 2008–2020

**Figure 1:**
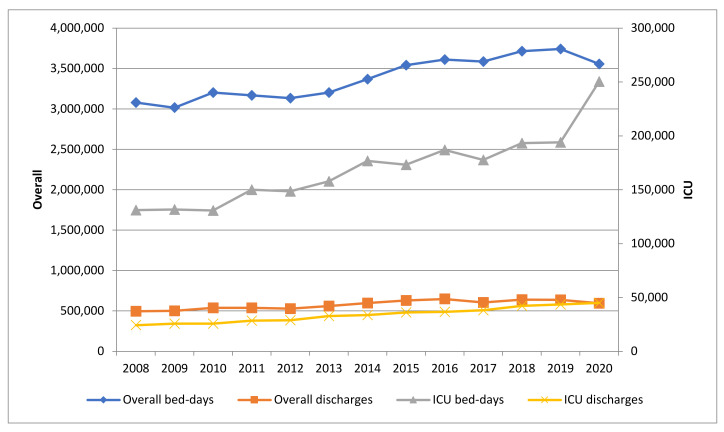
Evolution of overall and intensive care unit (ICU) bed-days and discharges 2008–2020.

The number of hospitals participating in the VINCat-PROA rose from 46 in 2008 to 66 in 2020 throughout the study period. These figures represented 68.8% and 89.7% of all adult acute hospital beds in Catalonia at these two time points. The number of recorded bed-days increased from 2,991,053 in 2008 to 3,557,047 in 2020, whereas the number of discharges increased from 497,306 in 2008 to 595,103 in 2020 (Figure 1).

Between 2019 and 2020, the number of participating hospitals increased from 64 to 66. The number of overall bed-days decreased by 0.05% (3,741,936 vs. 3,557,047 bed-days), and the number of discharges decreased by 0.07% (637,243 vs. 595,103 discharges). Regarding ICUs, the number of bed-days increased by 29.08% (194,011 vs. 250,425 bed-days) and the number of discharges increased by 3.24% (43,610 vs. 45,024 discharges). The hospitals added in 2020 were two small hospitals. Between them, they accounted for 22.662 bed-days and 5.365 overall discharges (0.6% and 0.9% of the total, respectively). With respect to the ICU, they accounted for 775 bed-days and 115 discharges (0.3% and 0.2% of the total, respectively).

The median number of overall and ICU days per patient in 2020 increased by 2.93% (5.8 vs. 5.97 days) and by 24.94% (4.45 vs. 5.56 days), respectively.

### 2.3. Changes in Global Consumption of Anti-Infectives for Systemic Use 2019–2020

The overall consumption of antibacterials in 2020 increased, compared to 2019, by 2.31% for DDD/100 bed-days (69.38 vs. 70.99; *p* < 0.001) and by 4.15% for DDD/100 discharges (407.41 vs. 424.30; *p* < 0.001) (Table 1).

This increase is mainly due to an increase in the consumption of macrolides and third-generation cephalosporins. It has been observed both in the consumption, expressed in DDD/100 bed-days (105.40%, *p* < 0.001 and 25.72%; *p* < 0.001, respectively), and in the consumption, expressed in DDD/100 discharges (109.08%, *p* < 0.001 and 27.97%; *p* < 0.001, respectively). A statistically significant decrease in the consumption of penicillins, aminoglycosides, and quinolones was also observed. The consumption of antifungals also increased significantly during 2020, both in DDD/100 bed-days (10.25%; 3.11 vs 3.43; *p* < 0.001) and in DDD/100 discharges (12.22%; 18.24 vs. 20.47; *p* < 0.001).

### 2.4. Changes in ICU Consumption of Anti-Infectives for Systemic Use 2019–2020

The variation in consumption in 2020 compared to 2019 in ICUs showed important differences, depending on whether it was calculated in DDD/100 bed-days or in DDD/100 discharges (Table 1 and Table 2).

The consumption of antibacterials, expressed in DDD/100 bed-days, decreased by 1.28% in 2020 (114.23 vs. 112.77; *p* < 0.001), mainly due to a decrease in the consumption of penicillins (−16.78%; *p* < 0.001), especially amoxicillin and beta-lactamase inhibitor (−29.42%; *p* < 0.001) and quinolones (−12.39%; *p* < 0.001). Likewise, the consumption of antimycotics, expressed in DDD/100 bed-days, in 2020 also decreased with respect to 2019 (12.35 vs. 11.81; −4.35%; *p* < 0.001), especially due to the decrease in the consumption of fluconazole (7.19 vs. 6.17; −14.12%; *p* < 0.001).

On the contrary, the consumption of antibacterial use, expressed in DDD/100 discharges, in 2020 increased by 23.42%, compared to 2019 (508.12 vs. 627.21; *p* < 0.001). This rise was observed in the main antibiotic families, although it has been especially important in macrolides (especially azithromycin 275.09%; *p* < 0.001), third- and fourth-generation cephalosporins (mainly ceftriaxone 55.11%; *p* < 0.001 and cefepime 106.35%; *p* < 0.001, respectively) and the “J01X other antibacterials” group (principally vancomycin 29.81%; *p* < 0.001 and linezolid 31.28%, *p* < 0.001). The consumption of antimycotics in 2020 per DDD/100 discharges increased, compared to 2019, by 19.58% (54.93 vs. 65.68; *p* < 0.001), primarily due to an increase in the consumption of amphotericin B (87.98%; *p* < 0.001) and voriconazole (96.17%, *p* < 0.001).

### 2.5. Comparison between Estimated and Observed ICU Consumption, Expressed in DDD/100 Discharges, in 2020

**Figure 2:**
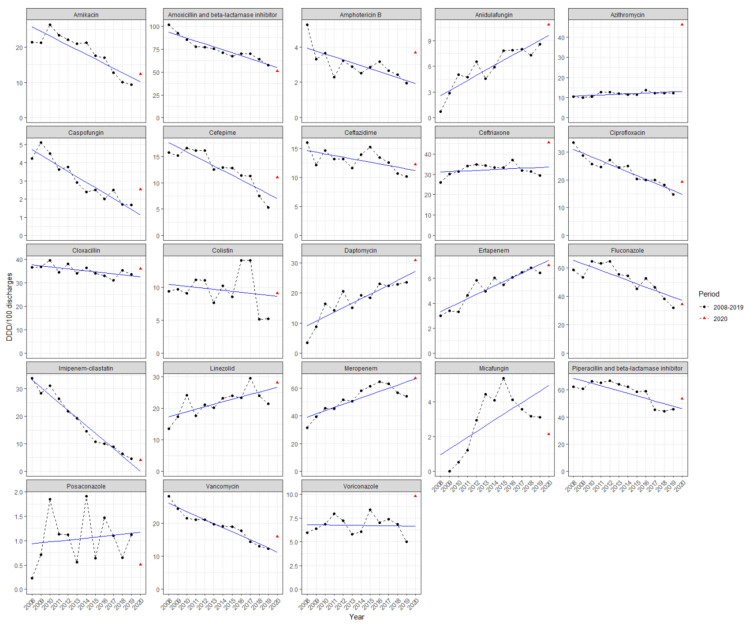
Evolution of antibiotic and antimycotic consumption in intensive care unit (ICU) services between 2008 and 2020, expressed in DDD/100 discharges. Non-COVID-19 period was considered from 2008 to 2019. DDD: Defined daily doses. Black circles: Antimicrobial consumption data for every year. Blue line: Antimicrobial consumption trend based on data from 2008 to 2019, and projected until 2020. Red triangle: Observed 2020 consumption.

Figure 2 shows the annual consumption in ICUs, expressed in DDD/100 discharges, of the different antibacterials and antimycotics for systemic use during the non-COVID-19 period (from 2008 to 2019), as well as the consumption trend line based on these data, projected until 2020.

The observed consumption of antibacterials in 2020 was 627.21 DDD/100 discharges. This consumption is above the estimated value (545.48 DDD/100 discharges) and exceeds the prediction interval (PI) based on the non-COVID-19 consumption trend from 2008 to 2019 (475.41 to 615.56 DDD/100 discharges). The 2020 consumption of antimycotics was 65.68 DDD/100 discharges, slightly higher than the estimated value (62.78 DDD/100 discharges), although within the PI (42.31 to 83.25 DDD/100 discharges).

Observed consumptions of amphotericin B, azithromycin, caspofungin, ceftriaxone, vancomycin, and voriconazole were higher than estimated consumption values and above the calculated PI (Table 3). These data, expressed in DDD/100 bed-days, have been included in the Supplementary Materials as Figure S1 and Table S1.

## 3. Discussion

The effect of the COVID-19 pandemic was to produce an imbalance in the use of antimicrobials, probably due to an initial poor knowledge of the evolution of the disease and to the interruption of the regular work of PROA.

In this study, including data from all the 66 hospitals of the VINCat program during the COVID-19 pandemic, antimicrobial consumption increased significantly, mainly in the ICU. The increase in some molecules, such as ceftazidime/avibactam, ceftolozane/tazobactam, colistin, amphotericin B, and voriconazole, deserves further study, and the implementation of strict antibiotic policies to optimize their use is necessary. Finally, when ICU data were expressed in DDD/100 bed-days compared to DDD/100 discharges, it yielded different consumption results.

One important finding of our study was the difference in ICU antimicrobial consumption observed for some molecules depending on the denominator (bed-days or discharges). The importance of describing both indicators was already described by VINCat in other non-COVID-19 settings, but has never been studied in COVID-19 or other pandemics [19]. Overall bed-days and discharges remained stable, which may explain the absence of differences when reporting consumption data. On the contrary, ICU bed-days increased by 29.08% and discharges by 3.24%. This may clarify the differences in some molecules, such as ertapenem, imipenem, or fluconazole, where DDD/100 bed-days decreased while DDD/100 discharges increased. The prolongation of hospital stay in ICUs has been essential in our results. Bearing in mind that consumption, expressed in DDD/100 discharges, allows us to approximate the antibiotic consumption made by each patient during their admission, these results indicate that, while antibiotic pressure per ICU day has changed relatively little (−1.28%), antibiotic pressure per ICU patient during the COVID-19 pandemic has increased greatly (23.42%). All these findings underline the importance of correlating clinical findings with consumption data, and makes difficult the comparison with previous non-pandemic periods.

Concerning microbiological data, we took as a reference the National Surveillance Study of Nosocomial Infection in the ICU (ENVIN-HELICS) registry [20]. This study was conducted from March 2020 to May 2020, including 1525 patients admitted to 61 ICUs from 54 hospitals in Spain. This work reflects the reality of Spanish ICUs during the first wave of the COVID-19 pandemic and brings into question some findings observed in our work.

An increase in overall antimicrobial consumption was shown, with worrisome results in ICUs. Based on Table 3, six molecules exceeded the PI: amphotericin B, caspofungin, voriconazole, ceftriaxone, azithromycin, and vancomycin.

This work is the first to report an important increase in antifungal consumption in ICUs. The rise in the incidence of invasive aspergillosis and candidemia during the COVID-19 pandemic could account for this finding [20,21]. This augmented risk is motivated by the SARS-CoV-2 infection itself, the prolonged ICU stay, exposure to broad-spectrum antibiotics, the development of acute respiratory distress syndrome, or the use of immunosuppressive therapies such as corticosteroids or interleukin-6 inhibitors including tocilizumab [21,22,23,24,25]. The higher risk of invasive aspergillosis may explicate the increment of voriconazole and amphotericin B consumption (which may have also been used as a nebulized therapy in intubated patients), although this polyene may have also been employed in the setting of fluconazole- and echinocandin-resistant candidiasis [25]. Echinocandins could have also been used as part of a combination therapy for invasive aspergillosis, although the rise in candidemia essentially explains these results. The use of fluconazole did not rise in parallel, which may reflect the lack of antifungal de-escalation or a higher incidence of fluconazole-resistant strains such as *Candida* spp. Compared to 2019 candidemia incidence in Spain, a higher incidence of *C. albicans* (7.1 vs. 5.5%) was observed, whereas *C. glabrata* (1.47 vs. 1.57%) and C. *parapsilosis* (2.94 vs. 2.36%) remained stable [20]. Concerning fluconazole resistance, it increased compared to the previous year: *C. albicans* (2/18, 11.1% vs. 0/13, 0%), *C. parapsilosis* (2/6, 33.3% vs. 1/8, 12.5%). As far as catheter-associated urinary tract infection (CAUTI) is concerned, a higher incidence of *C. albicans* (13.2 vs. 11.2%), *C. glabrata* (6.4 vs. 3.3%), and *C. parapsilosis* (3.2 vs. 1.4%) was observed. Despite this increase in the incidence and resistance of candida infections, microbiology does not support a higher use of these extended spectrum molecules in Spain [20].

The management of fungal infections in ICUs is a challenge, even more so in the context of COVID-19 [26]. Some PROA actions include a reduction in the length of therapy when possible, dose adjustments (i.e., not exceeding the use of 3 mg/kg of liposomal amphotericin B for invasive aspergillosis, instead of higher doses that have been proven to be deleterious [27]), therapeutic drug monitoring, intravenous to oral transition programs, or antifungal de-escalation [26].

Similarly, antibiotic consumption increased over this period. A higher incidence (8.1–9.3%) of bacterial co-infection was demonstrated in critically ill patients [2,22]. Therefore, given the severity of patients’ health and the greater mortality found in co-infected patients, empirical antimicrobial therapy was recommended in patients admitted to ICUs [12]. These can justify the astonishing increment in the consumption of ceftriaxone and azithromycin.

A higher consumption of drugs normally used for nosocomial infections, such as cefepime or meropenem, may be the consequence of a higher incidence of nosocomial infections [20]. As far as ventilator-associated pneumonia is concerned, *Pseudomonas aeruginosa* was the main involved (32.5%) microorganism in Spain [20]. Compared to the previous year, the resistance profile of this microorganism did not change, with similar resistance rates of meropenem, cefepime, or piperacillin/tazobactam [20]. However, a worrisome increase in the prevalence of carbapenem-resistant *Klebsiella pneumoniae* was observed [20]. The increase in the consumption of amikacin and ciprofloxacin, as part of combination therapy recommended by guidelines, may also be related [22,28,29]. The rise in the consumption of these molecules probably contributed, among other factors, to a higher incidence of *Clostridioides difficile* infection, which increased from 0.14% to 0.20%.

The rate of catheter-related bacteremia and CAUTI also increased [20]. A higher prevalence of enterococci (*Enterococcus faecium:* from 3.59% to 6.41% and *Enterococcus faecalis:* from 4.94% to 13.29%) was observed for both infections, probably due to the excessive use of cephalosporins [22]. The incidence of infections due to methicillin-resistant *S. aureus* also increased (from 1.35% to 1.98%). Due to these findings, the consumption of daptomycin, linezolid, and vancomycin was augmented.

A striking rise in the prescription of ceftolozane/tazobactam and ceftazidime/avibactam was observed, as previously described in Spain [15]. This result deserves further study, but data from the ENVIN-HELICS study highlighted that colonization/infection by multidrug-resistant (MDR) organisms during ICU stay was more frequent during the COVID-19 pandemic. This finding was especially important for extended spectrum beta-lactamases (7.5 vs. 1.8%), MDR *P. aeruginosa* (4.7 vs. 0.6%), and carbapenemase producing organisms (4.4 vs. 0.4%).

On the other hand, the use of amoxicillin/clavulanate practically disappeared, which may also reflect the lack of antimicrobial de-escalation or a shift towards a greater use of ceftriaxone.

Beyond microbiology, from a global perspective, several reasons may also account for these results. Firstly, the SARS-CoV-2 pandemic has radically changed the paradigm of what we have known so far about viral infections in the last 50 years. Patients with COVID-19 differ from those diagnosed with influenza, with an up to three-fold higher in-hospital mortality, higher overburdened ICU capacity, ICU admission (16.3% vs. 10.8%, *p* < 0.0001), ICU mean length of stay (15 vs. 8 days, *p* < 0.0001), need for mechanical ventilation (71.5% vs. 61.0%, *p* < 0.0001), and ICU mortality (27.1% vs. 18.0%, *p*< 0.0001) [22,30,31].

Secondly, other global challenges were faced: non-ICU healthcare workers not familiar with infection prevention and control principles (IPCP) were admitted to ICUs, unfamiliarity with ICU complications, increased workload, PROA meeting suspension, use of unfamiliar tools and equipment, decreased attention to IPCP, shortages of personal protective equipment, and less invasive microbiological sampling due to concerns of generated aerosols [32].

Our study yielded important and worrisome results. The epidemic of MDR bugs is one of the biggest challenges of modern medicine, and the SARS-CoV-2 pandemic has only aggravated it [33]. In the light of further COVID-19 waves and potential pandemics causes by other viruses, it is essential to learn from past mistakes and try to optimize future processes. PROA should persist and be actively involved in the management of these situations [34]. To try to reduce the unnecessary antibiotic consumption in the setting of the COVID-19 pandemic, different strategies have been proposed, as well as recommendations of research areas to strengthen PROA teams [8,32,35,36].

This study is not without limitations. Unfortunately, as we expressed the annual consumption data, we were not able to discern the impacts of the different waves (and, therefore, variants). Likewise, we did not study the indications of the antimicrobials, nor their duration, if they were empirical or directed, and the isolated microbiology. The lack of microbiological data is an important limitation to study the potential justification of prescribed antibiotics. In addition, the lack of this information together with the observational nature of the study precludes the demonstration of a temporary association between antibiotic exposure and resistance. We acknowledge that an analysis of the impact of the different types of hospitals would have been of interest, but this was out of our scope. Finally, we recognize that the use of DDD may not be the best indicator in an ICU setting, where higher or reduced doses can be employed compared to pre-defined standard doses [37]. This finding may be more evident during the COVID-19 pandemic, where a higher prevalence of obesity or need for mechanical ventilation and renal replacement therapies was observed [31,32]. Although days of therapy is the recommended indicator by the WHO, they are not always available.

## 4. Materials and Methods

### 4.1. Setting and Study Design

All acute care hospitals affiliated with the VINCat program [16] participated in this retrospective, longitudinal, and descriptive study.

### 4.2. Data Collection

The anatomical therapeutic chemical/DDD (ATC/DDD), system developed and updated by the World Health Organization (WHO) Collaborating Centre for Drug Statistics Methodology has become an international standard for drug metrics and facilitates the presentation and comparison of drug consumption statistics at international, national, and regional levels. In 2008, the VINCat-PROA adopted the ATC/DDD system as a standardized measure.

The ATC/DDD system was used to monitor adult hospital anti-infectives for systemic use consumption every year. The pharmacy departments at the participating hospitals reported the number of units of each antibacterial for systemic use (J01) and antimycotic for systemic use (J02) dispensed from the whole hospital, from the medical and surgical units, and from the ICUs. Bed-days data and discharge data were also informed. The admission and discharge days were considered as a single day [19].

Adult antibacterial and antimycotic consumption were calculated for each year as DDD/100 bed-days and DDD/100 discharges with the WHO ATC/DDD index 2021.

All hospitals received appropriate training before starting the study and on an annual basis by the VINCat coordinating center, to guarantee an homogeneous collection of the data according to pre-established criteria [16]. Afterwards, all the registered information was assessed and validated.

### 4.3. Statistical Analysis

Differences in antibacterial and antimycotic consumption, expressed in DDD/100 bed-days and DDD/100 discharges, between 2019 and 2020 were analyzed using the exact rate ratio test, achieving their corresponding confidence intervals (CIs) and *p*-values for each comparison.

Estimated non-COVID-19 2020 antibiotic and antimycotic ICU consumption was estimated by a simple linear regression model, considering only the period 2008–2019 in the predictor variable. Observed ICU 2020 consumptions were compared with non-COVID-19 2020 estimated ICU consumptions, and the corresponding PI were obtained to compare them with their respective observed 2020 ICU consumptions.

For all statistical analysis, 95% CIs and PIs were calculated. *p*-values of <0.05 were considered statistically significant. A bilateral distribution was assumed for all *p*-values. The analyses were performed using R v4.0.4 statistical software.

## 5. Conclusions

In summary, the consumption indicators for most antimicrobials have deviated from the expected trend pattern based on the information available from previous years. The greater increase in DDD/100 discharges reflects a higher individual exposure to antimicrobials, with the consequent risk of altering the microbiota of patients admitted to VINCat hospitals during the COVID-19 pandemic. Future studies should assess the real impact of these findings.

## Figures and Tables

**Table 1:** Changes in overall and intensive care unit (ICU) consumption of anti-infectives for systemic use, 2019–2020.

			DDD/100 Bed-Days				DDD/100 Discharges			
Service	ATC Classification		2019	2020	Variation (%)	*p*	2019	2020	Variation (%)	*p*
**Overall**	**J01 Antibacterials for systemic use**		69.38	70.99	2.31	<0.001	407.41	424.30	4.15	<0.001
	**J01C Penicillins**		23.63	21.70	−8.18	<0.001	138.76	129.69	−6.53	<0.001
		J01CR Combinations of penicillins. incl. beta-lactamase inhibitors	18.26	16.80	−7.98	<0.001	107.23	100.44	−6.33	<0.001
	**J01DBCDE Cephalosporins**		14.10	15.19	7.78	<0.001	82.78	90.82	9.71	<0.001
		J01DD Third-generation cephalosporins	7.56	9.51	25.72	<0.001	44.40	56.82	27.97	<0.001
		J01DE Fourth-generation cephalosporins	0.64	0.65	2.05	0.03	3.75	3.89	3.87	<0.001
	**J01DH Carbapenems**		5.01	5.16	2.93	<0.001	29.41	30.82	4.77	<0.001
	**J01DI Other cephalosporins and penems**		0.29	0.37	29.00	<0.001	1.68	2.20	31.32	<0.001
	**J01FA Macrolides**		3.26	6.69	105.40	<0.001	19.13	40.00	109.08	<0.001
	**J01G Aminoglycoside antibacterials**		1.82	1.63	−10.72	<0.001	10.69	9.71	−9.12	<0.001
	**J01M Quinolone antibacterials**		8.48	7.20	−15.01	<0.001	49.77	43.06	−13.49	<0.001
	**J01X Other antibacterials**		8.89	9.39	5.72	<0.001	52.18	56.15	7.61	<0.001
		J01XA Glycopeptide antibacterials	2.56	2.75	7.43	<0.001	15.01	16.42	9.36	<0.001
		J01XB Polymyxins	0.39	0.34	−12.96	<0.001	2.28	2.02	−11.40	<0.001
		J01XX Other antibacterials	3.70	4.01	8.45	<0.001	21.72	23.98	10.39	<0.001
	**J02A Antimycotics for systemic use**		3.11	3.43	10.25	<0.001	18.24	20.47	12.22	<0.001
		J02AB Imidazole derivatives	0.00	0.00	−81.25	<0.001	0.03	0.01	−80.99	<0.001
		J02AC Triazole derivatives	2.61	2.81	7.85	<0.001	15.31	16.81	9.78	<0.001
		J02AX Other antimycotics for systemic use	0.39	0.44	14.62	<0.001	2.27	2.64	16.67	<0.001
**ICU**	**J01 Antibacterials for systemic use**		114.23	112.77	−1.28	<0.001	508.18	627.21	23.42	<0.001
	**J01C Penicillins**		32.92	27.40	−16.78	<0.001	146.46	152.38	4.04	<0.001
		J01CR Combinations of penicillins. incl. beta-lactamase inhibitors	23.24	18.74	−19.35	<0.001	103.37	104.23	0.84	0.21
	**J01DBCDE Cephalosporins**		17.26	18.20	5.46	<0.001	76.80	101.26	31.84	<0.001
		J01DD Third-generation cephalosporins	12.11	13.78	13.82	<0.001	53.87	76.66	42.30	<0.001
		J01DE Fourth-generation cephalosporins	1.20	1.98	65.04	<0.001	5.35	11.03	106.35	<0.001
	**J01DH Carbapenems**		14.66	14.02	−4.32	<0.001	65.20	77.99	19.62	<0.001
	**J01DI Other cephalosporins and penems**		0.87	1.19	36.09	<0.001	3.88	6.60	70.13	<0.001
	**J01FA Macrolides**		7.62	13.60	78.44	<0.001	33.90	75.62	123.09	<0.001
	**J01G Aminoglycoside antibacterials**		4.11	3.88	−5.64	<0.001	18.30	21.58	17.97	<0.001
	**J01M Quinolone antibacterials**		9.33	8.18	−12.39	<0.001	41.52	45.48	9.54	<0.001
	**J01X Other antibacterials**		19.34	19.67	1.69	0.01	86.04	109.38	27.13	<0.001
		J01XA Glycopeptide antibacterials	5.41	5.65	4.49	<0.001	24.07	31.45	30.64	<0.001
		J01XB Polymyxins	1.17	1.64	40.27	<0.001	5.20	9.11	75.36	<0.001
		J01XX Other antibacterials	10.46	10.82	3.40	<0.001	46.55	60.18	29.28	<0.001
	**J02A Antimycotics for systemic use**		12.35	11.81	−4.35	<0.001	54.93	65.68	19.58	<0.001
		J02AB Imidazole derivatives	0.00	0.00	-	-	0.00	0.00	-	-
		J02AC Triazole derivatives	8.91	8.36	−6.17	<0.001	39.62	46.48	17.31	<0.001
		J02AX Other antimycotics for systemic use	3.00	2.79	−6.96	<0.001	13.36	15.54	16.33	<0.001

ICU: Intensive care unit; DDD: Defined daily doses; ATC: Anatomical therapeutic chemical.

**Table 2:** Changes in intensive care unit (ICU) consumption of major anti-infectives for systemic use 2019–2020.

	ICU DDD/100 Bed-Days				ICU DDD/100 Discharges			
ATC Classification	2019	2020	Variation (%)	*p*	2019	2020	Variation (%)	*p*
J02AA01 Amphotericin B	0.44	0.66	50.34	<0.001	1.95	3.67	87.98	<0.001
J01GB06 Amikacin	2.12	2.22	4.91	0.02	9.43	12.37	31.16	<0.001
J01CR02 Amoxicillin and beta-lactamase inhibitor	12.94	9.13	−29.42	<0.001	57.58	50.81	−11.76	<0.001
J02AX06 Anidulafungin	1.93	1.95	1.40	0.52	8.57	10.86	26.77	<0.001
J01FA10 Azithromycin	2.77	8.32	200.01	<0.001	12.34	46.28	275.09	<0.001
J02AX04 Caspofungin	0.38	0.45	20.13	<0.001	1.68	2.52	50.18	<0.001
J01DE01 Cefepime	1.20	1.98	65.04	<0.001	5.35	11.03	106.35	<0.001
J01DI02 Ceftaroline fosamil	0.43	0.31	−28.21	<0.001	1.91	1.71	−10.25	0.03
J01DD02 Ceftazidime	2.28	2.20	−3.89	0.05	10.17	12.21	20.16	<0.001
J01DD52 Ceftazidime and beta-lactamase inhibitor	0.33	0.58	77.11	<0.001	1.46	3.23	121.47	<0.001
J01DI54 Ceftolozane and beta-lactamase inhibitor	0.44	0.88	98.22	<0.001	1.97	4.89	147.81	<0.001
J01DD04 Ceftriaxone	6.61	8.19	24.07	<0.001	29.38	45.58	55.11	<0.001
J01MA02 Ciprofloxacin	3.31	3.47	4.72	0.01	14.73	19.28	30.92	<0.001
J01CF02 Cloxacillin	7.56	6.45	−14.69	<0.001	33.62	35.86	6.66	<0.001
J01XB01 Colistin	1.17	1.64	40.27	<0.001	5.20	9.11	75.36	<0.001
J01XA04 Dalbavancin	0.00	0.00	57.14	0.68	0.00	0.01	96.67	0.53
J01XX09 Daptomycin	5.26	5.53	5.13	<0.001	23.42	30.78	31.44	<0.001
J01DH03 Ertapenem	1.45	1.27	−12.83	<0.001	6.46	7.04	8.99	<0.001
J02AC01 Fluconazole	7.19	6.17	−14.12	<0.001	31.98	34.34	7.37	<0.001
J01DH51 Imipenem-cilastatin	1.02	0.70	−31.62	<0.001	4.54	3.89	−14.50	<0.001
J02AC05 Isavuconazole	0.34	0.29	−14.33	<0.001	1.52	1.63	7.10	0.20
J01XX08 Linezolid	4.81	5.06	5.01	<0.001	21.42	28.12	31.28	<0.001
J01DH02 Meropenem	12.18	12.06	−1.02	0.24	54.19	67.06	23.75	<0.001
J02AX05 Micafungin	0.70	0.39	−44.67	<0.001	3.10	2.14	−30.81	<0.001
J01CR05 Piperacillin and beta-lactamase inhibitor	10.29	9.61	−6.68	<0.001	45.79	53.43	16.68	<0.001
J02AC04 Posaconazole	0.25	0.09	−63.53	<0.001	1.12	0.51	−54.39	<0.001
J01XA01 Vancomycin	2.76	2.87	3.83	0.04	12.30	15.97	29.81	<0.001
J02AC03 Voriconazole	1.12	1.76	56.92	<0.001	4.99	9.80	96.17	<0.001

ICU: Intensive care unit; DDD: Defined daily doses; ATC: Anatomical therapeutic chemical.

**Table 3:** Comparison between expected and observed intensive care unit (ICU) consumption, expressed in DDD/100 discharges. Estimated 2020 consumption was based on the trend from the non-COVID-19 period (2008–2019).

	ICU DDD/100 Discharges			
	Estimated 2020 Consumption	Observed 2020 Consumption	Variation (%)	Prediction Intervals
J02AA01 Amphotericin B	1.93	3.67	90.16	[0.28 to 3.57]
J01GB06 Amikacin	10.17	12.37	21.63	[3 to 17.34]
J01CR02 Amoxicillin and beta-lactamase inhibitor	54.96	50.81	−7.55	[43.43 to 66.49]
J02AX06 Anidulafungin	9.64	10.86	12.66	[6.77 to 12.52]
J01FA10 Azithromycin	13.03	46.28	255.18	[10.66 to 15.41]
J02AX04 Caspofungin	1.14	2.52	121.05	[0.05 to 2.23]
J01DE01 Cefepime	7.02	11.03	57.12	[2.84 to 11.19]
J01DD02 Ceftazidime	11.22	12.21	8.82	[7.28 to 15.16]
J01DD04 Ceftriaxone	33.59	45.58	35.70	[25.9 to 41.29]
J01MA02 Ciprofloxacin	14.78	19.28	30.45	[10.21 to 19.35]
J01CF02 Cloxacillin	32.39	35.86	10.71	[27.45 to 37.33]
J01XB01 Colistin	8.65	9.11	5.32	[0.98 to 16.32]
J01XX09 Daptomycin	27.08	30.78	13.66	[18.92 to 35.24]
J01DH03 Ertapenem	7.46	7.04	−5.63	[6.05 to 8.88]
J02AC01 Fluconazole	37.17	34.34	−7.61	[20.93 to 53.4]
J01DH51 Imipenem-cilastatin	0.0	3.89	NA	[−5.22 to 5.01]
J01XX08 Linezolid	26.73	28.12	5.20	[18.44 to 35.03]
J01DH02 Meropenem	66.79	67.06	0.40	[51.71 to 81.87]
J02AX05 Micafungin	4.93	2.14	−56.59	[1.28 to 8.58]
J01CR05 Piperacillin and beta-lactamase inhibitor	46.21	53.43	15.62	[32.52 to 59.9]
J02AC04 Posaconazole	1.17	0.51	−56.41	[−0.23 to 2.57]
J01XA01 Vancomycin	11.32	15.97	41.08	[7.97 to 14.67]
J02AC03 Voriconazole	6.65	9.80	47.37	[4.03 to 9.27]

ICU: Intensive care unit; DDD: Defined daily doses.

## Data Availability

The data presented in this study are available on request from the corresponding author.

## Supplementary Materials

The following are available online at https://www.mdpi.com/article/10.3390/antibiotics10080943/s1. Figure S1: Evolution of antibiotic and antimycotic consumption in ICU services between 2008 and 2020, expressed in DDD/100 bed-days. Non-COVID-19 period was considered from 2008 to 2019. Table S1: Comparison between expected and observed intensive care unit (ICU) consumption, expressed in DDD/100 bed-days. Estimated 2020 consumption was based on the trend from the non-COVID-19 period (2008–2019). File S1: Contribution-Log.
