# Supplementary material for: Antimicrobial Consumption among 66 Acute Care Hospitals in Catalonia: Impact of the COVID-19 Pandemic
Source: Antibiotics (Basel). 2021 Aug 4;10(8):943. doi: 10.3390/antibiotics10080943 (PMC8388964; doi:10.3390/antibiotics10080943)
Supplement: Supplementary file 1 [file antibiotics-10-00943-s001.zip › antibiotics-1311261-File S1. Contribution log.pdf]

File S1. Contribution log

Collaborators list:

Ana **Ayestaran**: Department of Pharmacy, Hospital de Barcelona, 08034 Barcelona, Spain; aayestarana@gmail.com
Ana **Montero**: Department of Pharmacy, Hospital Universitario Quiron Dexeus, 08028 Barcelona, Spain; ana.montero@quironsalud.es
Anisi **Moron**: Department of Pharmacy, Hospital de Sabadell, 08208 Sabadell, Spain; Amoron@tauli.cat
Anna **Capellà**: Department of Pharmacy, Parc Sanitari S. Joan Déu - HG, 08830 Sant Boi de Llobregat, Spain; anna.capella@pssjd.org
Anna **Clopes**: Department of Pharmacy, Institut Català d'Oncologia, 08908 L'Hospitalet de Llobregat, Spain; aclopes@iconcologia.net
Antònia **Balet**: Department of Pharmacy, Althaia, Hospital de Sant Joan de Déu, 08243 Manresa, Spain; abalet@althaia.cat
Ariadna **Padulles**: Department of Pharmacy, Hospital Universitari de Bellvitge, 08907 L'Hospitalet de Llobregat, Spain; apadulles@bellvitgehospital.cat
Begoña **Pascual**: Department of Pharmacy, Hospital Municipal de Badalona, 08911 Badalona, Spain; bpascual@bsa.cat
Camil la **Valls**: Department of Pharmacy, Hospital Universitari de Vic, 08500 Vic, Spain; cvalls@chv.cat
Carme **Ortiz**: Department of Pharmacy, Centre MQ Reus, 43201 Reus, Spain; carme.ortiz@centremq.cat
Carmen **Muñoz**: Department of Pharmacy, ICO L'Hospitalet, 08908 L'Hospitalet de Llobregat, Spain; cms@iconcologia.net
Cristina **Toro**: Department of Pharmacy, Hospital Santa Caterina, 17190 Salt, Spain; cristina.toro@ias.cat
Daniel **Serrano**: Department of Pharmacy, Hospital El Pilar, 08006 Barcelona, Spain; daniel.serrano@quironsalud.es
Daria **Ayago**: Department of Pharmacy, Hospital Comarcal d'Amposta, 43870 Amposta, Spain; daria.ayago@grupsagessa.com
David **Brandariz**: Department of Pharmacy, Hospital QuirónSalud Barcelona, 08023 Barcelona, Spain; vrandariz@gmail.com
David **Campany**: Department of Pharmacy, Hospital Universitari Vall d'Hebron, 08035 Barcelona, Spain; dcampany@vhebron.net
Eduard **Hidalgo**: Department of Pharmacy, H. de l'Hospitalet-H. Moisès Broggi, 08970 Sant Joan Despí, Spain; eduard.hidalgo@sanitatintegral.org
Edurne **Fernandez**: Department of Pharmacy, Hospital de la Santa Creu i Sant Pau, 08041 Barcelona, Spain; eFernandezG@santpau.cat
Elisenda **Flotats**: Department of Pharmacy, Hospital de la Cerdanya, 17520 Puigcerdà, Spain; eflotats@hcerdanya.eu
Esperanza **Gil**: Department of Pharmacy, Hospital Comarcal Móra d'Ebre, 43770 Móra d'Ebre, Spain; esperanza.gil@grupsagessa.cat
Esther **Julián**: Department of Pharmacy, Hospital de Tortosa Verge de la Cinta, 43500 Tortosa, Spain; ejulian.ebre.ics@gencat.cat
Esther **López**: Department of Pharmacy, Hospital Clínic de Barcelona, 08036 Barcelona, Spain; eslopez@clinic.cat
Eugènia **Òdena**: Department of Pharmacy, Hospital Comarcal de Sant Bernabé, 08600 Berga, Spain; eodena@hsb.cat
Gemma **Enrique-Tarancon**: Department of Pharmacy, Clínica Nova Aliança, 25006 Lleida, Spain; genriquetarancon@minovalianca.com
Glòria **Gayola**: Department of Pharmacy, Clínica Girona, 17002 Girona, Spain; farmaceutics@clinicagirona.cat
Isabel **Frigola**: Department of Pharmacy, Clínica Salus Infirmorum, 17820 Banyoles, Spain; ifrigola@clinicasalus.org
Isabel **Martínez**: Department of Pharmacy, Hospital Sant Pau i Santa Tecla and Hospital del Vendrell, 43003 Tarragona, Spain; imartinez@xarxatecla.cat
Jordi **Fernández**: Department of Pharmacy, Centre Mèdic Teknon, 08022 Barcelona, Spain; jfernandez@cmteknon.com

Josep **Torrent**: Department of Pharmacy, Pius Hospital de Valls, 43800 Valls, Spain; jtorrent@piushospital.cat
Juan **Serrais**: Department of Pharmacy, Hospital d'Igualada, 08700 Igualada, Spain; jserrais@csa.cat
Laura **Canadell**: Department of Pharmacy, H Univ. Joan XXIII de Tarragona, 43005 Tarragona, Spain; lcanadell.hj23.ics@gencat.cat
Laura **Gratacos**: Department of Pharmacy, Hospital U Dr Josep Trueta de Girona, 17007 Girona, Spain; lgratacos.girona.ics@gencat.cat
Leonor **Munell**: Department of Pharmacy, Hospital de Campdevànol, 17530 Campdevànol, Spain; lmunell@hoscamp.com
Lidia **Salse**: Department of Pharmacy, Fundació Puigvert - IUNA, 08025 Barcelona, Spain; lsalse@fundacio-puigvert.es
Ludivina **Ibañez**: Department of Pharmacy, Espitau Val d'Aran, 25530 Vielha, Spain; libanez@aransalut.com
Mª José **Fraile**: Department of Pharmacy, Hospital Sant Rafael, 08035 Barcelona, Spain; mjfraile.hsrafael@hospitalarias.es
Mª Rosa **Garriga**: Department of Pharmacy, Hospital U. Mútua de Terrassa, 08221 Terrassa, Spain; rgarriga@mutuaterrassa.es
Maite **Barrera**: Department of Pharmacy, Hospital Dos de Maig, 08025 Barcelona, Spain; mariateresa.barrerapuigdollers@sanitatintegral.org
Manuel **Cano**: Department of Pharmacy, Hospital U. Arnau de Vilanova de Lleida, 25198 Lleida, Spain; smcano.lleida.ics@gencat.cat
Maria **Alcaide**: Department of Pharmacy, ICO Badalona, 08916 Badalona, Spain; malcalder@iconcologia
Maria **José Berges**: Department of Pharmacy, Centre Prevenció i Rehabilitació Asepeyo, 08010 Barcelona, Spain; mbergesfraile@asepeyo.es
Maria **Sagales**: Department of Pharmacy, Hospital General de Granollers, 08402 Granollers, Spain; msagales@fhag.es
Marina **De Temple**: Department of Pharmacy, Hospital Universitari Sagrat Cor, 08029 Barcelona, Spain; mdetemple@quironsalud.es
Marisa **Grañó**: Department of Pharmacy, Hospital Comarcal del Pallars, 25620 Tremp, Spain; mgranyo@hospitalpallars.com
Marlene **Alvarez**: Department of Pharmacy, H. U. Germans Trias i Pujol de Badalona, 08916 Badalona, Spain; malvarezm.germanstrias@gencat.cat
Marta **Martí**: Department of Pharmacy, Hospital Sant Joan de Déu (Martorell), 08760 Martorell, Spain; mmarti@hmartorell.es
Melisa **Barrantes**: Department of Pharmacy, Hospital Plató, 08006 Barcelona, Spain; mebarrantes@clinic.cat
Mireia **Fuster**: Department of Pharmacy, Hospital de Terrassa, 08227 Terrassa, Spain; mfuster@cst.cat
Montse **Lladó**: Department of Pharmacy, Centre Mèdic Delfos, 08023 Barcelona, Spain; llado.far@centromedicodelfos.es; llado.far@delfos.cat
Montserrat **Carrascosa**: Department of Pharmacy, Clínica Terres de l'Ebre, 43500 Tortosa, Spain; montserrat.carrascosa@saluttortosa.cat
Montserrat **Navarro**: Department of Pharmacy, Hospital Santa Maria, 25198 Lleida, Spain; mnavarro@gss.cat
Natàlia **Carrasco**: Department of Pharmacy, Hospital de Viladecans, 08840 Viladecans, Spain; ncarrasco.hv@gencat.cat
Nuri **Quer**: Department of Pharmacy, ICO Girona, 17007 Girona, Spain; nquer@iconcologia.net
Núria **Bosacoma**: Department of Pharmacy, Hospital de Palamós, 17230 Palamós, Spain; nbosacoma@ssibe.cat
Núria **Miserachs**: Department of Pharmacy, Fundació Hospital de l'Esperit Sant, 08923 Santa Coloma de Gramenet, Spain; nmiserac@fhes.cat
Olga **Curiel**: Department of Pharmacy, Hospital de Sant Celoni, 08470 Sant Celoni, Spain; ocuriel@hsceloni.cat
Patricia **Domínguez**: Department of Pharmacy, Hospital de Mollet, 08100 Mollet del Vallès, Spain; p.dominguez@fsm.cat
Pilar **Alemany**: Department of Pharmacy, Hosp. d'Olot i Comarcal de la Garrotxa, 17800 Olot, Spain; palemany@hospiolot.com
Pilar **Salvador**: Department of Pharmacy, Hospital Universitari Sant Joan de Reus, 43204 Reus, Spain; psalvador@grupsagessa.com

Rafael **Alberti**: Department of Pharmacy, Hospital Comarcal de Blanes, 17300 Blanes, Spain; ralberti@salutms.cat
Sandra **Barbadillo**: Department of Pharmacy, Hospital General de Catalunya, 08195 Barcelona, Spain; sbarbadillo@quironsalud.es
Silvia **Serdà**: Department of Pharmacy, Hospital Residència Sant Camil, 08810 Sant Pere de Ribes, Spain; sserda@csg.cat
Susana **Terré**: Department of Pharmacy, HC Sant Jaume Calella i HC de Blanes, 08370 Calella, Spain; sterre@salutms.cat
Teresa **Arranz**: Department of Pharmacy, Hospital Comarcal de l'Alt Penedès, 08720 Vilafranca del Penedès, Spain; tarranz@csap.cat
Teresa **Gurrera**: Department of Pharmacy, Hospital de Mataró, 08304 Mataró, Spain; tgurrera@csdm.cat
Usúe **Manso**: Department of Pharmacy, Fundació Sant Hospital, 25700 La Seu d'Urgell, Spain; farmacia@fsh.cat
Virginia **Gol**: Department of Pharmacy, Hospital de Figueres, 17600 Figueres, Spain; vgol@salutemporda.cat
Virginia Maria **Martinez**: Department of Pharmacy, Institut Guttmann, 08916 Badalona, Spain; vmmartinez@guttmann.com

Collaborators Contributions: Data curation, A.A., A.M. (Ana Montero), A.M. (Anisi Moron), A.C. (Anna Capellà), A.C. (Anna Clopes), A.B., A.P., B.P., C.V., C.O., C.M., C.T., D.S., D.A., D.B., D.C., E.H., E.F. (Edurne Fernandez) ,E.F. (Elisenda Flotats),E.G., E.J., E.L., E.Ò., G.E.-T., G.G., I.F., I.M., J.F., J.T., J.S., L.C., L.G., L.M., L.S., L.I., M.J.F., M.R.G., M.B. (Maite Barrera), M.C. (Manuel Cano), M.A. (Maria Alcaide), M.J.B., M.S., M.D.T., M.G., M.A. (Marlene Alvarez), M.M., M.B. (Melisa Barrantes), M.F., M.L., M.C. (Montserrat Carrascosa), M.N., N.C., N.Q., N.B., N.M., O.C., P.D., P.A., P.S., R.A., S.B., S.S., S.T., T.A., T.G., U.M., V.G. and V.M.M.

Conflicts of interest: all the authors declare no conflicts of interest.
