# Supplementary material for: Antimicrobial Consumption among 66 Acute Care Hospitals in Catalonia: Impact of the COVID-19 Pandemic
Source: Antibiotics (Basel). 2021 Aug 4;10(8):943. doi: 10.3390/antibiotics10080943 (PMC8388964; doi:10.3390/antibiotics10080943)
Supplement: Supplementary file 1 [file antibiotics-10-00943-s001.zip › antibiotics-1311261-supplementary.pdf]

Supplementary Materials

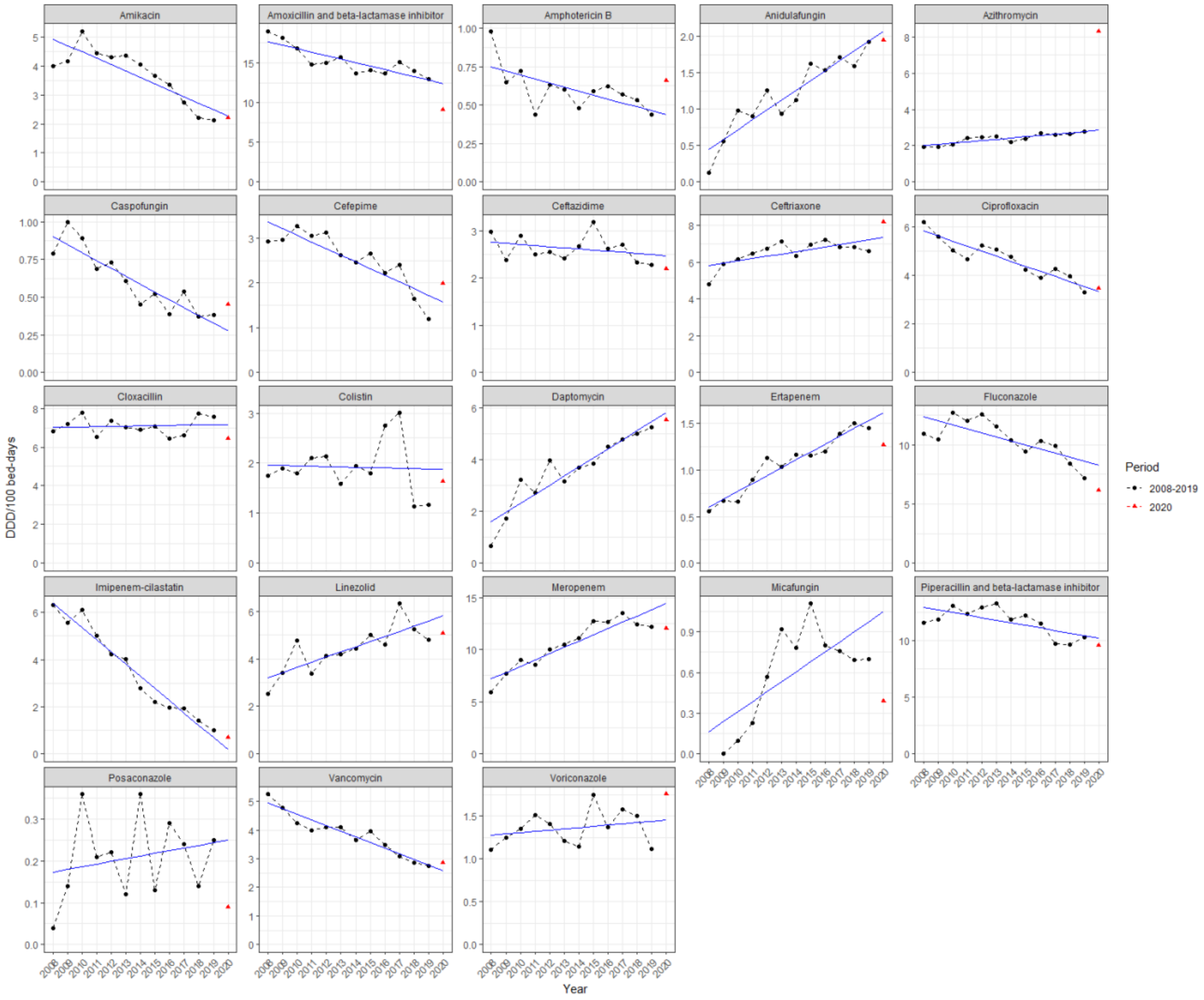

Figure S1. Evolution of antibiotics and antimycotics consumption in ICU services between 2008 and 2020 expressed in DDD/100 bed-days. Non COVID-19 period was considered from 2008 to 2019. *Black circles:* Antimicrobial consumption data for every year. *Blue line:* Antimicrobial consumption trend based on data from 2008 to 2019 and projected until 2020. *Red triangle*: Observed 2020 consumption.

Table S1. Comparison between expected and observed intensive care unit (ICU) consumption expressed in DDD/100 bed-days. Estimated 2020 consumption was based on the trend from the non-COVID-19 period (2008–2019).

	**ICU DDD/100 bed-days**			
	**Estimated 2020 Consumption**	**Observed 2020 Consumption**	**Variation (%)**	**Prediction intervals**
J02AA01 Amphotericin B	2.28	2.22	−2.63%	[0.89,3.67]
J01GB06 Amikacin	12.37	9.13	−26.19%	[9.65,15.09]
J01CR02 Amoxicillin and beta-lactamase inhibitor	0.44	0.66	50.00%	[0.13,0.74]
J02AX06 Anidulafungin	2.07	1.95	−5.80%	[1.55,2.6]
**J01FA10 Azithromycin**	**2.85**	**8.32**	**191.93%**	**[2.48,3.21]**
J02AX04 Caspofungin	0.27	0.45	66.67%	[0.02,0.53]
J01DE01 Cefepime	1.58	1.98	25.32%	[0.74,2.42]
J01DD02 Ceftazidime	2.48	2.2	−11.29%	[1.75,3.2]
J01DD04 Ceftriaxone	7.33	8.19	11.73%	[6.04,8.61]
J01MA02 Ciprofloxacin	3.34	3.47	3.89%	[2.51,4.18]
J01CF02 Cloxacillin	7.17	6.45	−10.04%	[5.92,8.41]
J01XB01 Colistin	1.87	1.64	−12.30%	[0.37,3.38]
J01XX09 Daptomycin	5.82	5.53	−4.98%	[4.41,7.22]
J01DH03 Ertapenem	1.61	1.27	−21.12%	[1.39,1.84]
J02AC01 Fluconazole	8.25	6.17	−25.21%	[5.32,11.17]
J01DH51 Imipenem-cilastatin	0.19	0.7	268.42%	[−0.85,1.23]
J01XX08 Linezolid	5.8	5.06	−12.76%	[4.1,7.5]
J01DH02 Meropenem	14.43	12.06	−16.42%	[11.95,16.92]
J02AX05 Micafungin	1.05	0.39	−62.86%	[0.33,1.76]
J01CR05 Piperacillin and beta-lactamase inhibitor	10.21	9.61	−5.88%	[7.7,12.73]
J02AC04 Posaconazole	0.25	0.09	−64.00%	[−0.01,0.51]
J01XA01 Vancomycin	2.57	2.87	11.67%	[1.96,3.18]
J02AC03 Voriconazole	1.46	1.76	20.55%	[0.92,1.99]

ICU: Intensive Care Unit; DDD: Defined daily doses.
